# Supplementary material for: A Comparison of Inertial Measurement Units and Overnight Videography to Assess Sleep Biomechanics
Source: Bioengineering (Basel). 2023 Mar 25;10(4):408. doi: 10.3390/bioengineering10040408 (PMC10136254; doi:10.3390/bioengineering10040408)

Supplementary Materials:

Table S1: Participant Instructions

Thank you for being part of our project! Please see below the instructions for how to collect data on your sleep movement. If you have any questions or issues, please don't hesitate to contact me at nicholas.buckley@telethonkids.org.au or on +61 8 6319 1771.

Step 1: Applying and starting the sensors

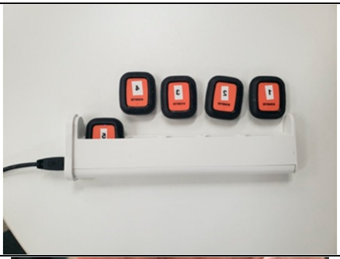	o Take sensors off charge<br>o Take the 4 DOTs off charge<br>o The mobile phone can stay on charge
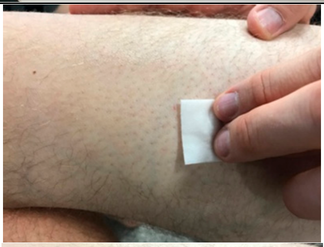<br>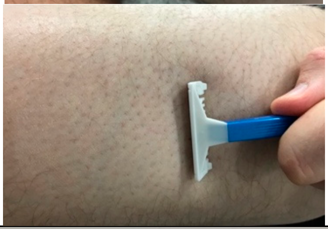	o Preparing the skin<br>o Use the provided alcohol wipe to remove any oil from the skin; this will help the dressing stick better<br>o If the area is hairy, use the provided disposable razor to remove the hair from the area (you don't have to, but this will make taking the dressings off much more comfortable!)
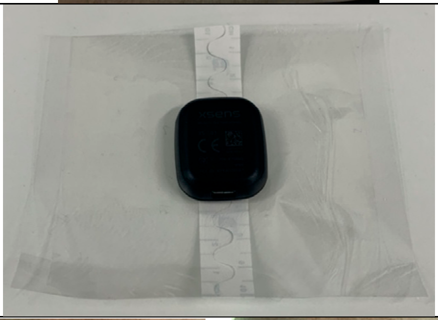	o Stick DOTs to Tegaderm<br>o Take the sticky backing off the Tegaderm dressing, and stick the FRONT of the DOT to the sticky surface of the dressing
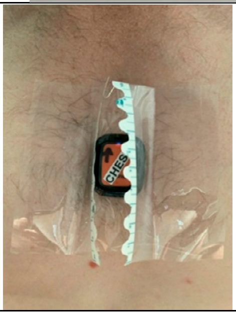 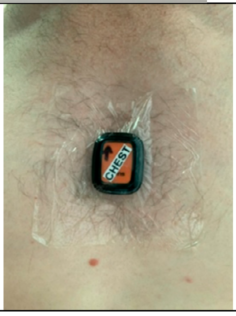	o Stick DOTs to body<br>o Stick the 4 DOTs on the spots located below-make sure they are pointing the right way up (arrow pointing towards head)

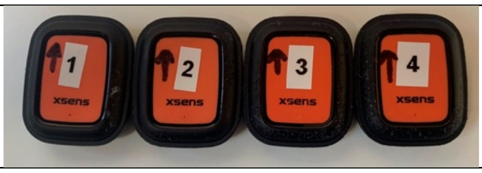	

Step 2: Recording with the App

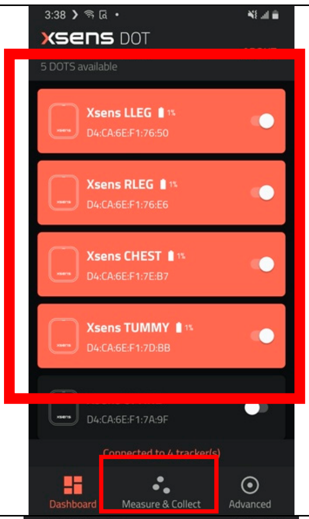	○ Open the XENS DOTs app, and make sure all the dots are switched on (switches on the right)<br>○ When you are ready to record, tap on the 'Measure and Collect' tab at the bottom of the screen
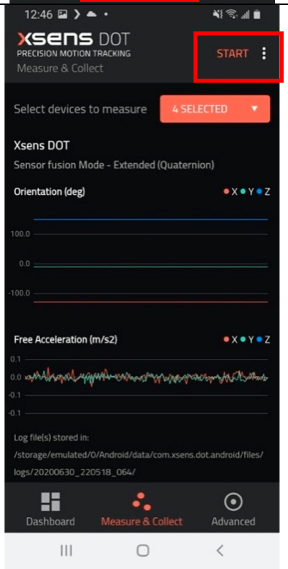	○ On the next screen, tap 'start' at the top right of the screen

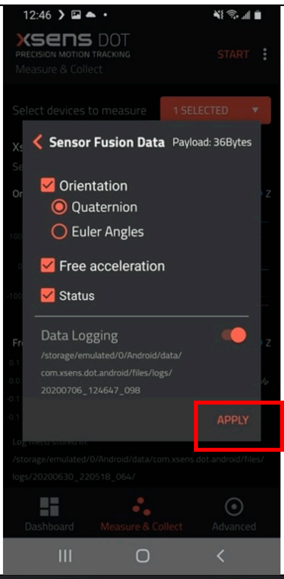	o All of the setting will be automatically applied, please don't change them<br>o Tap 'Apply' to start the recording
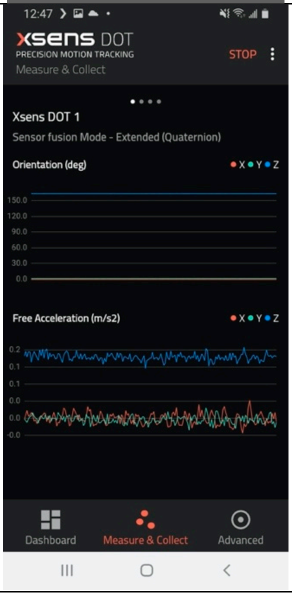	o You will now see your movements recorded on the screen – you have started recording!<br>o Make sure the phone is plugged in to power before you go to sleep<br>o You now can go to sleep – the sensors will automatically stop, so you don't need to do that<br>o If you need to get up during the night (e.g. go to the bathroom) that's ok – please just take the phone with you so the sensors stay in range (2-3 metres)

Step 3: Starting and stopping cameras

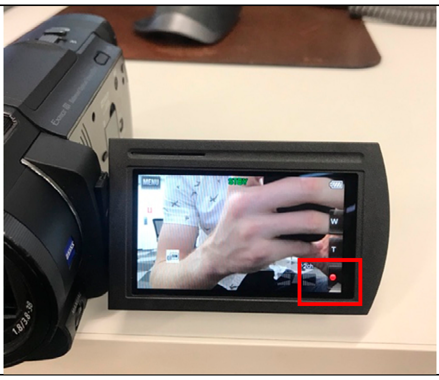	o Press 'record' on the screen of all three cameras to start them recording, then go to sleep as normal<br>o When you wake up, press the same 'record' button to stop the recording

Videos S1–S2: Training Videos for Raters

Video S1: BODS Rating Protocol Training Video

https://youtu.be/dGPkX5xFaFA

Video S2: BODS Introduction Video

https://youtu.be/qWXFVaVjJFc

Table S2: BODS Rating Form

Video Footage Rating Form

Rater Name:____________________________ Participant ID:___________________________ Date:___________________________________

	Starting Position				Ending Position			
	Sternum	Pelvis	L Leg	R Leg	Sternum	Pelvis	L Leg	R Leg
Clip 1								
Clip 2								
Clip 3								
Clip 4								
Clip 5								
Clip 6								
Clip 7								
Clip 8								
Clip 9								
Clip 10								

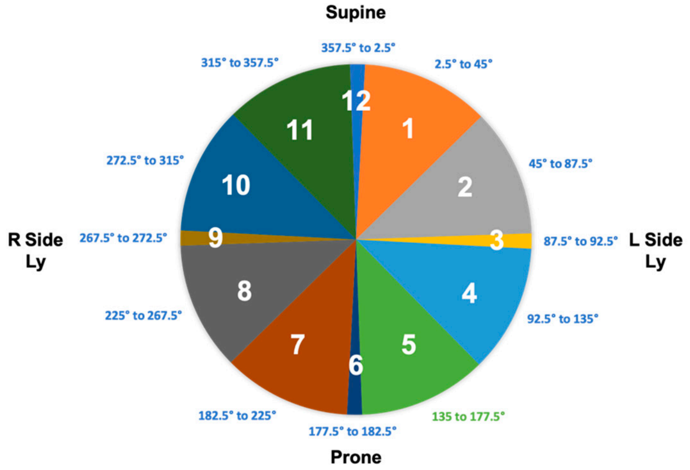

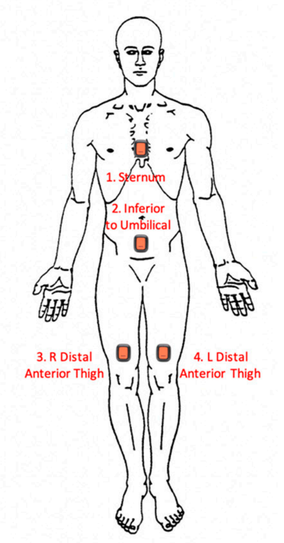

Supplement: Supplementary file 1 [file bioengineering-10-00408-s001.zip › bioengineering-2271099-supplementary.pdf]
